# Towards Human in the Loop Analysis of Complex Point Clouds: Advanced Visualizations, Quantifications, and Communication Features in Virtual Reality

**DOI:** 10.3389/fbinf.2021.775379

**Published:** 2022-01-20

**Authors:** Thomas Blanc, Hippolyte Verdier, Louise Regnier, Guillaume Planchon, Corentin Guérinot, Mohamed El Beheiry, Jean-Baptiste Masson, Bassam Hajj

**Affiliations:** ^1^ Laboratoire Physico-Chimie, Institut Curie, PSL Research University, CNRS UMR168, Paris, France; ^2^ Sorbonne Universités, UPMC Univ Paris 06, Paris, France; ^3^ Decision and Bayesian Computation, CNRS USR 3756, Department of Computational Biology and Neuroscience, CNRS UMR 3571, Université de Paris, Institut Pasteur, Université de Paris, Paris, France; ^4^ Sorbonne Universités, Collège Doctoral, Paris, France

**Keywords:** virtual reality, point clouds, inference, microscopy, human in the loop, 3D maps

## Abstract

Multiple fields in biological and medical research produce large amounts of point cloud data with high dimensionality and complexity. In addition, a large set of experiments generate point clouds, including segmented medical data or single-molecule localization microscopy. In the latter, individual molecules are observed within their natural cellular environment. Analyzing this type of experimental data is a complex task and presents unique challenges, where providing extra physical dimensions for visualization and analysis could be beneficial. Furthermore, whether highly noisy data comes from single-molecule recordings or segmented medical data, the necessity to guide analysis with user intervention creates both an ergonomic challenge to facilitate this interaction and a computational challenge to provide fluid interactions as information is being processed. Several applications, including our software DIVA for image stack and our platform Genuage for point clouds, have leveraged Virtual Reality (VR) to visualize and interact with data in 3D. While the visualization aspects can be made compatible with different types of data, quantifications, on the other hand, are far from being standard. In addition, complex analysis can require significant computational resources, making the real-time VR experience uncomfortable. Moreover, visualization software is mainly designed to represent a set of data points but lacks flexibility in manipulating and analyzing the data. This paper introduces new libraries to enhance the interaction and human-in-the-loop analysis of point cloud data in virtual reality and integrate them into the open-source platform Genuage. We first detail a new toolbox of communication tools that enhance user experience and improve flexibility. Then, we introduce a mapping toolbox allowing the representation of physical properties in space overlaid on a 3D mesh while maintaining a point cloud dedicated shader. We introduce later a new and programmable video capture tool in VR and desktop modes for intuitive data dissemination. Finally, we highlight the protocols that allow simultaneous analysis and fluid manipulation of data with a high refresh rate. We illustrate this principle by performing real-time inference of random walk properties of recorded trajectories with a pre-trained Graph Neural Network running in Python.

## Introduction

Scientific research is producing large amounts of data of various types and dimensionality. Exploring this increasingly complex data is essential to guide experiments, rapidly extract relevant information, and guide future developments. Multidimensional point cloud data are generated in several scientific fields, including LIDAR, computer-assisted design, segmented medical images, and electron microscopy, to name a few. This paper shows an application centered on point clouds generated from single-molecule localization microscopy (SMLM) (Betzig et al., 2006; Rust et al., 2006) without losing the generality of the proposed method solutions.

In SMLM, single-molecules are observed in their natural cellular environment to extract information related to their dynamics and interactions in live cells (Höfling and Franosch, 2013; Manzo and Garcia-Parajo, 2015), or to reconstruct super-resolution images of cell organelles (Betzig et al., 2006; Rust et al., 2006). Large-scale recordings of single molecules at the nanometer resolution reveal the complex interplay between 4D geometry (space and time) and biological activity. Understanding these complex time-evolving structures requires freely navigating within the data and analyzing portions of it.

Another significant component associated with single-molecule imaging is the complexity of the data. Positions and dynamics of biomolecules are accessed through multiple preliminary operations (Chenouard et al., 2014), such as image deconvolution and tracking, which leads to noisy datasets. Furthermore, these datasets are usually highly heterogeneous in space, time, and other associated properties. Furthermore, organelles unseen in datasets influence the geometry of the recorded point cloud in the surrounding environment. Hence, we are convinced that numerous scientific initiatives benefit from immersive visualization modalities such as virtual reality (VR) to enhance data comprehension. Data in this context may be uploaded to a VR environment and visualized using a custom shader program linked to relevant experimental parameters.

Exploring the data in VR offers the possibility to grasp the global geometrical structures while allowing the possibility to dive within the data to explore more local structures and their relation in space. Besides, the interaction in VR facilitates the quantitative analysis of the data through the combination of user intervention and optimized analysis algorithms. The ability to easily and quickly isolate regions of interests in 3D for example is time consuming and impractical even with efficient desktop software such as ViSP (Beheiry and Dahan, 2013), while these sorts of interactions are faster and more precise using VR. Several applications including our software platform Genuage are dedicated to visualize and analyze point cloud data in virtual reality (Blanc et al., 2020). Genuage is an open-source VR–compatible platform for visualization of n-dimensional point cloud data. It features a set of VR-assisted tools for data exploration, data selection, and built-in analysis. While VR visualization parameters can be adapted to the various types and dimensionality of the recorded point clouds, quantification, on the other hand, seems far from being standard. Point clouds generated in different acquisition modalities can vary in dimensionality and might underlay specific information that requires dedicated algorithms for analysis. Moreover, such algorithms are developed in different scripting platforms and remain case-dependent. The standardization of the quantification process remains thus difficult. In addition, complex analysis can require large computational resources, making the real-time VR experience uncomfortable. Moreover, visualization software is mostly designed to represent a set of data points but lacks flexibility in manipulating and analyzing the data. Examples include generating new data properties to visualize, calculating physical properties from selected points, creating new point clouds derived from the one already uploaded and combining data sets.

This paper presents the Genuage software for point cloud visualization and interaction and highlights recent developments. We introduce new approaches and processes implemented within the Genuage platform, in which enhanced flexibility and user experience are made possible by a set of communication tools and built-in functions. We discuss communication protocols with user-defined libraries applied on a selection or an entire data set. This communication mode allows DLLs to compute specific properties on selected data, generate new data sets or create a new point cloud. We demonstrate this approach by analyzing the blinking of single-molecule localization data where it is desirable to combine localizations originated from blinking molecules, to compute local density, or to determine dynamical properties. We introduce a new set of tools for calculating and presenting physical properties in 3D overlaid to a 3D mesh while maintaining a point cloud dedicated shader. We additionally introduce learning procedures on graphs and their relation to points clouds in a biological context. We show the interaction with data performed while maintaining a high refresh rate within the VR headsets, thus maintaining user comfort when communicating with other scripting platforms even if large computational resources are required. We illustrate this principle by performing real-time inference of random walk properties of recorded trajectories with a pre-trained Neural Network running in Python. Finally, we introduce a new programmable video capture tool in VR and desktop modes for intuitive data dissemination.

## Methods

### VR for Point Cloud Visualization

VR is starting to play a more important role in research initiatives. From cardboard inserts intended for smartphones to advanced headsets with multiple sensors for positional tracking, VR contributes to the design of projects for various science topics. Facilitating this process are freely accessible development platforms, such as Unity and Unreal Engine, and the availability of many open-source projects. VR applications can now be a part of the teaching (Brůža et al., 2021) and research environments (Matthews, 2018; El Beheiry et al., 2019).

Applications centered on point clouds span various fields. Some relate to data tagging with machine learning such as those in (Bergé et al., 2016; Stets et al., 2017; Wirth et al., 2019; Liu et al., 2020; Ramirez et al., 2020). They range from optimized interfacing to easy interactions with the data to mixed interactions with various algorithms to prepare data or detect specific structures of interest. Some hybrid applications can be found in astronomy such as (Gaia Sky, 2014). General visualization and interaction software include PointCloud XR and developments centered on compression to ensure visualization in VR (and AR) (Pavez et al., 2018). Other applications such as MegaMol and UnityMol are dedicated for the visualization of point-based molecular data sets (Doutreligne et al., 2014; Grottel et al., 2015). Within the context of microscopy and biological data analysis, which is the focus of this paper, the open source vLUME software (Spark et al., 2020) provides a direct way to represent, interact, and analyze static point clouds from single-molecule experimental data. It features numerous interfaces and high-quality visualization to highlight structures and better understand biomolecule geometric distributions within the cell. We provide in the supplementary a detailed feature comparison table.

### Genuage for Point Cloud Representation and Analysis

#### General Features of Genuage

**FIGURE 1:**
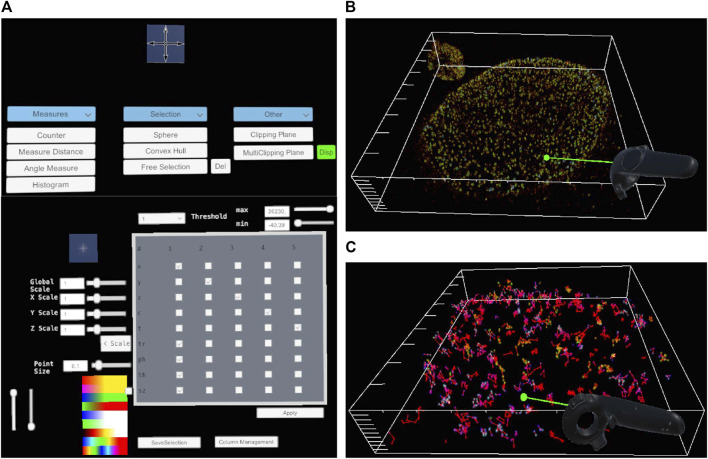
General overview of Genuage. The VR interface **(A)** allows the adjustment of the visual parameters associated with the point cloud. It includes associating visual parameters to specific columns in the data, adjusting the color code, scaling, and aspect ratio. A set of analysis tools allow performing VR-assisted measurements and complicated 3D selections. Clipping plane tools allow us to explore the inner layers of the point cloud in VR at arbitrary angles. Multidimensional point cloud data generated by single-molecule localization microscopy are explored in VR in 3D **(B)** and (3D + time) **(C)**.

We recently introduced the open-source software platform Genuage (Blanc et al., 2020; Genuage GitHub, 2020) to address visualization, interaction and analysis of complex multi-dimensional point cloud data. The platform can accommodate several types of point cloud data. Genuage is built on the Unity game engine and was initiated on the simple premise that extracting information from high dimensional point clouds is a non-trivial task. VR immersion provides the dual possibility of increasing the number of dimensions represented and allowing more direct interaction with data. Genuage has a dual interface: a computer desktop interface and a VR interface. The desktop interface derives its design principle from ViSP (Beheiry and Dahan, 2013): it allows users to load the data and set general representation parameters (Supplementary Figure S2). While all these parameters could be defined within the VR interface, data visualization and analysis practices in the lab are often incompatible with long times passed within the VR environment. Hence, we developed a dynamic process where the user can switch from one interface to the other based on the specificity and complexity of the action, they wish to execute following the same design principles of DIVA (El Beheiry et al., 2020). The VR interface allows visualization and navigation within the point cloud (Figure 1; Supplementary Figure S3). It provides multiple means of interaction and for adjusting visualization parameters within the VR environment. In addition, various tools for measuring distances, angles, counting, and time series analysis are accessible.

The data sets and associated parameters presented in this paper stems from single-molecule localization microscopy experiments and present some specificities that we will highlight in the following paragraphs.

#### Single-Molecule Experiments and Data

Fluorescence microscopy is a powerful tool in modern biological studies. It provides a window to observe the inner functioning of cell organelles in physiological conditions with high specificity. In conventional fluorescence microscopy, images of the whole sample are acquired with a limited resolution due to the optical diffraction limit (Lichtman and Conchello, 2005). However, imaging individual molecules allow overcoming this limit. Individual labeled proteins can be imaged and localized with high precision in live and fixed cells thanks to bright fluorescing molecules. Imaging sparse distributions of single molecules over time and localizing their centers with high precision provides two types of information. First, it offers a means to follow the dynamics of individual emitters in live cells. The quantification of each molecule’s diffusion behavior and interaction kinetics provides a statistical map of molecular interactions avoiding thus population averaging. Second, it allows the reconstruction of super-resolution images with a resolution below the diffraction limit of the microscope. The principle relies on splitting the emission of molecules in time and space such that only a sparse subset of molecules is active in a single acquisition frame. Each molecule is localized with a precision way below the diffraction limit. Repeated steps of localization, bleaching, and activation of a new subset of single-molecules allow to sample the whole structure of interest and reconstruct a pointillist image in super-resolution. In both cases, the output of the localization algorithm is a set of 3D coordinates [e.g., (Hajj et al., 2014; Nehme et al., 2020)], and, depending on the complexity of the detection microscope, several associated parameters such as the color, the intensity of the detected molecule, the time stamp, and the molecular orientation to name a few. Visualizing and analyzing such complex datasets is not trivial on a 2D screen given the large dimensionalities of the recorded point cloud. The complexity and the density of data points can conceal or intersect with other features within the data. In addition to the visualization aspects, analysis might require specific tasks to be performed within the complex dataset such as selection and counting. Users often face the need to analyze a specific trajectory or a selection of trajectories within a region of interest. Thus, the main requirements for exploiting single-molecule localization data are an efficient and intuitive perception of multidimensional data, ease of navigation through the data, and a facilitated interaction to accomplish specific tasks such as placing landmarks for measurements or counting and performing selections.

There is a clear advantage in using Genuage and VR to navigate and interact with such complex data. However, analyses of single-molecule datasets are far from being standard. It is common for different users to adopt different strategies for the quantification of the recorded cloud. Custom-made algorithms are often required and can continuously improve with evolving functionalities. Moreover, different algorithms might impact the original point cloud differently, for instance by generating additional properties for the point cloud or by creating a completely new data set. There is thus a clear need to adapt Genuage for different custom-made algorithms with non-standardized outputs.

#### General Framework for Visualization and Analysis in the Genuage Platform

A table of 3D coordinates defines the point clouds and other associated parameters generated by a specific experimental and analysis approach. Genuage can read point cloud data from any text file with any separator type providing that the data is organized in rows and disregarding the presence of any header. Individual points are represented directly with geometric shaders as Gaussian shapes with adjustable standard deviations to provide an adequate “size” to the points within the VR environment. The geometry shader spawns 4 points per data point to define a sprite with the Gaussian shape. Characteristics of Gaussian profiles such as color, intensity, or transparency can be mapped to specific data columns associated with a set of point cloud parameters or obtained as a result of performed analysis. Different color code palettes, including colorblind friendly palettes, are provided to account for different experimental data requirement and avoid visualization artifact (Borland and Taylor Ii, 2007). Subsets of points are visible or invisible based on specific thresholding parameters with nearly no impact on software performance or framerate. In addition, other high-dimensional information can be presented, such as vectorial data associated with points. For example, molecular orientations appear as an overlay on the point cloud of individual orientation-dependent color-coded segments. Similarly, we can display dynamic (time series) point clouds as a function of the recorded time or scrolling time windows. Trajectories are linked via segments in 3D, as can be seen in (Figure 1C). The user fully controls the mapping and correspondence between data columns and visual parameters. In (Figure 1A) we show a few components of the interface that are directly related to the discussed application.

Visualizing dense 3D point clouds can be challenging as the user’s relative position concerning the observed reconstructed data may not fully visualize the underlying structure. Similarly, highly anisotropic data can be challenging to visualize due to orientation dependency. In Genuage, the user can render a set of point cloud data visible or invisible based on thresholding specific parameters present in the data. Also, thanks to a clipping plane feature, the user can control within the VR environment a 3D plane that conceals the points it intersects. Furthermore, this clipping plane can be held in a place and combined with several additional ones to allow exploration of dense point cloud data at arbitrary angles.

In addition to the visualization aspects, we provide several built-in quantification tools in Genuage. VR is a vehicle to perform otherwise time-consuming quantification tasks more precisely. A set of essential tools include 3D length measurement, counting, angle measurement, profiling the point cloud along a segment as a bin count, selecting a subset of the point cloud, and exporting data. All geometric operations in 3D space are simplified thanks to the VR environment. JSON files are generated as a means to save the manipulation progress and to record the analysis results.

Advanced analysis such as diffusion coefficient inference on selected time series data complements the essential tools that are presented. In addition, a new tool to overlay different shaders is implemented to represent local physical properties computed on the local point cloud data set. For example, we provide an analysis tool for creating a complex 3D mesh based on local point cloud density. In each region, the code infers the local diffusion behavior of dynamic molecules data and presents it as a variation in the color of the mesh or as surfaces with adequate color. The implementation will be further detailed in the following section.

**FIGURE 2:**
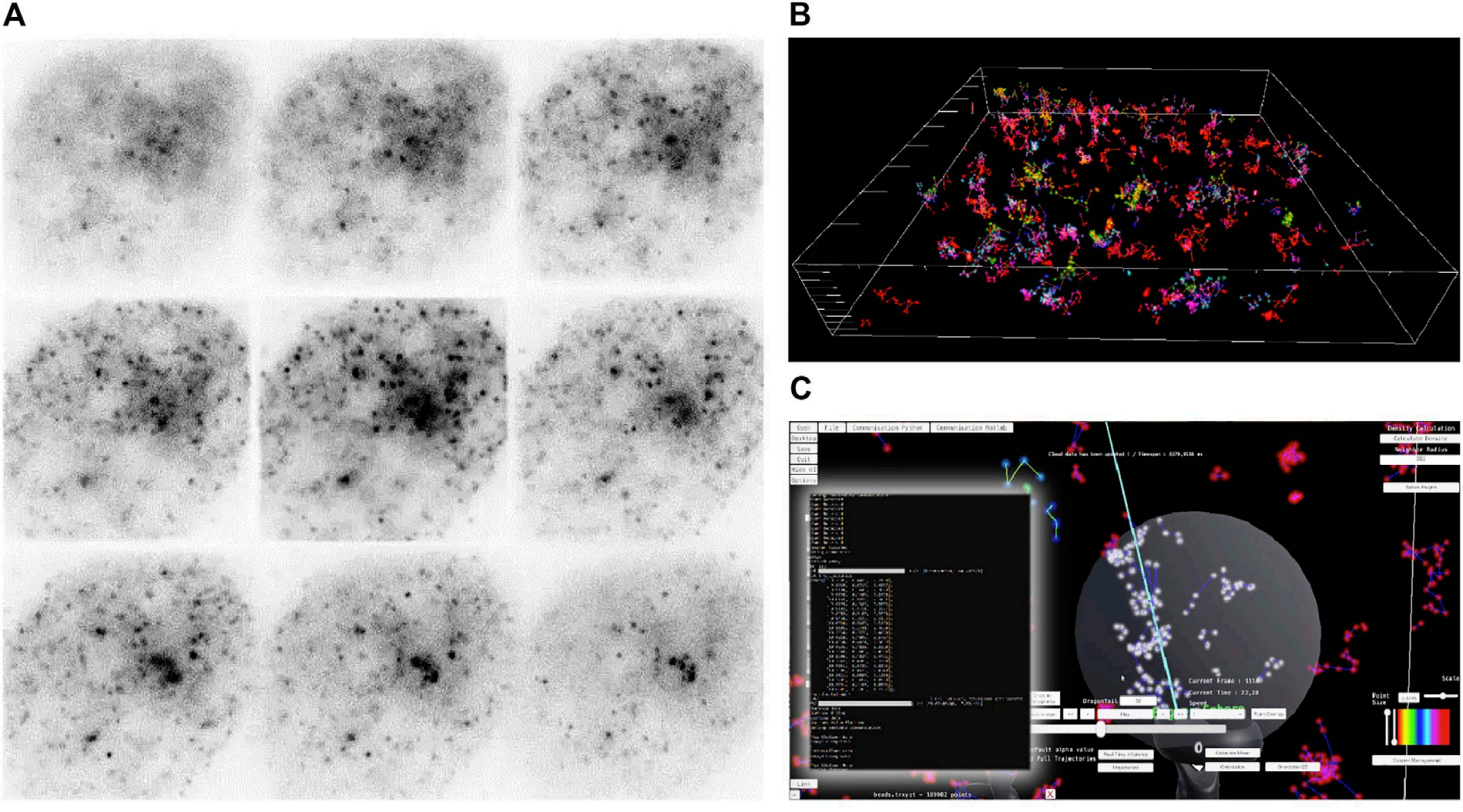
Workflow for single-molecule analysis in Genuage. **(A)** Raw images of diffusing particles in the nucleus of a cell are captured with MFM. Images are analyzed, and a table is generated containing the position of each particle and the corresponding timestamp. **(B)** The localizations are imported into Genuage and explored in VR. Color codes can be tuned to represent individual trajectories or the temporal evolution of the point cloud dynamics. **(C)** A screenshot of the Genuage interface as a selection of point cloud subsets and analysis is performed. Trajectory selection is performed using the VR controllers with a visual aid in the form of a sphere. Other shapes can be used to select points. In highly convoluted point distributions, the user can define landmarks generating a convex hull that will capture points of interest. Results are displayed as changes of the trajectory colors, with intensity depending on the value of the sub-diffusive exponent. The inference analysis is performed in real-time on the selected trajectories (inset shows real-time calculation performed). Various means to display results are available. The screenshot also shows the interface in the current version of the Genuage software. Most of the buttons and displays are self-explanatory [an introduction to the design can be found in the videos associated with (Blanc et al., 2020)].

The Genuage platform is developed as a versatile package for point cloud data and is highly modular to allow easy repurposing of portions of the code. Besides visualization, the goal of Genuage is efficient, and simplified data analysis. As point cloud data is ubiquitous, developing a general analysis framework is challenging. Our first development ensured compatibility with Python with a dedicated communication protocol launchable from Genuage. This development was motivated by ensuring that complex analysis could be performed outside the platform, especially if they required significant computational resources. We used a similar approach for volumetric data annotation in the DIVA-cloud platform (Voxel Learning in DIVA, 2020). Efficient uses of the pipeline consist of transmitting a subset of selected data to a python script for analysis and using the results to enhance the visualization of this subset or provide extra information in space and time. We also implemented the Matlab pipeline as it is a popular tool for biological data analysis. Similar pipelines will soon be added for R and Julia. An example of an application is shown using a custom Graph neural network to perform single-molecule trajectory analysis (see Figure 2; Supplementary Video S1).

**FIGURE 3:**
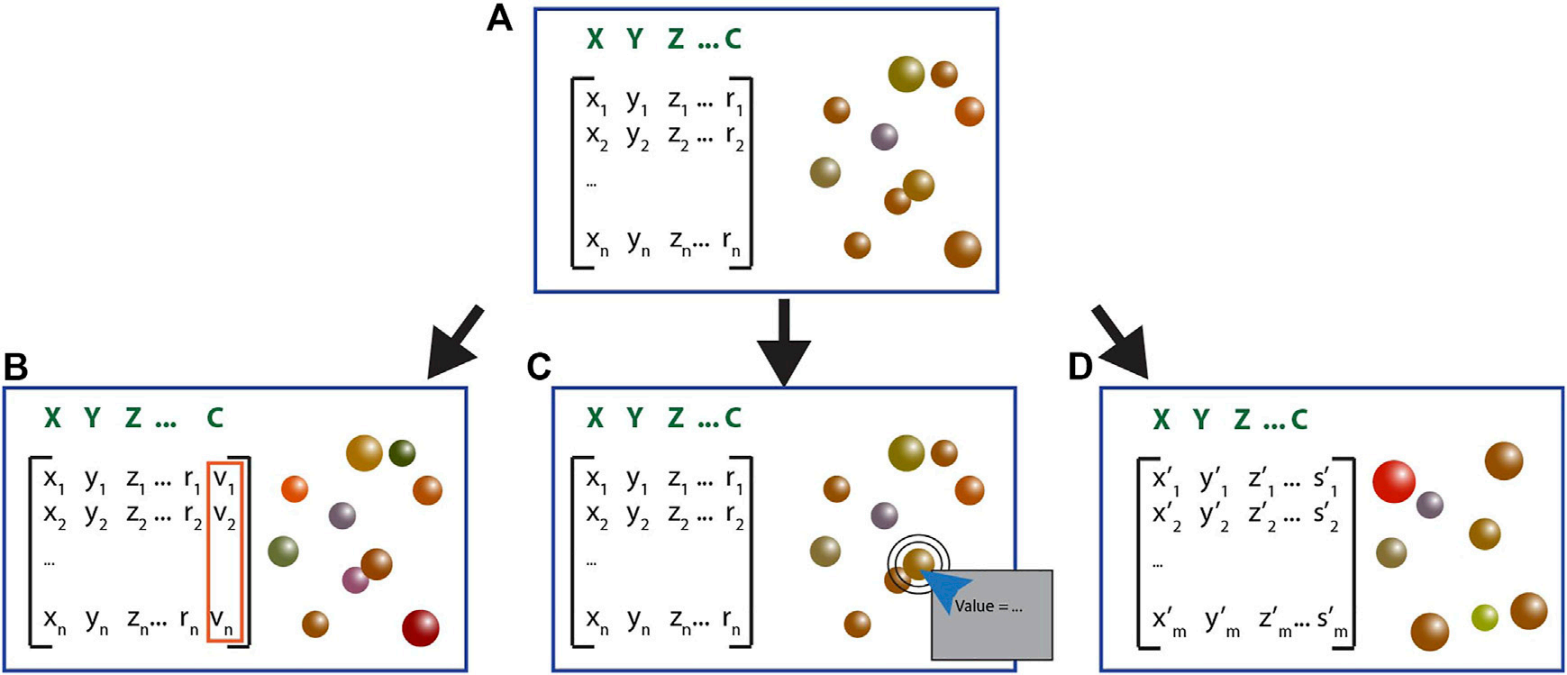
Different library configurations are compatible with Genuage and allow performing specific manipulation of the data. **(A)** Initially, the point cloud is imported as a table of coordinates displays in Genuage as 3D spheres positioned at (X,Y,Z) coordinates with specific visual attributes like colors (c). Depending on its format, the DLL: **(B)** performs a specific calculation on the point cloud that results in an additional data column that could be assigned to a specific visual feature, **(C)** retrieves the information from selected points. It displays the resulting calculus in a popping up window, **(D)** generates an entirely new point cloud with different elements based on specific mathematic manipulation.

Our newest development focused on numerical data analysis that either requires limited computational resources or requires a complex set of packages. We added a toolbox to import ad-hoc external DLLs libraries. While we use the classical development process of using addons, plugins, and DLLs to enhance the Genuage platform, our challenge lied in the end-user community willingness to engage in the challenges of developing VR software and in their limited computing facilities (usually computers with basic graphic cards and few CPU cores). We streamlined the development and use of a VR software for experimental scientist and biologists. Furthermore, the architecture is optimized to ensure usability on most computing platforms. We provide different templates for the required possible formats of the DLLs and structures. Different DLL formats allow performing specific tasks. Currently, we provide 3 configurations of libraries that allow efficient integration within the Genuage platform (Figure 3; Supplementary Video S2). These configurations provide guidelines for a user to develop other possible integration architectures. We provide code templates to help user easily create DLLs from their analysis algorithms, and integrate their code into an analysis routine through Genuage. Users are able to run custom algorithms with no need for advanced knowledge in C# or software development. We favored three analysis configurations. First, the library generates new columns in the point cloud data represented within the VR environment and associated to a specific visual parameter (Figure 3B). Second, the library generates numerical outputs shown in an extra visualization window (Figure 3C). Third, the library creates a new point-cloud dataset (Figure 3D). To illustrate these procedures and provide development guidelines for new libraries, we provide three DLLs. The first one simplifies point density estimation. The second one computes the apparent diffusion coefficient on a selected trajectory. The third one combines and displays point clouds localizations generated by the same molecule due to the inherent molecular blinking commonly encountered in single-molecule super-resolution acquisitions (check Supplementary Information for more details).

While VR is instrumental for data analysis, results are often communicated without the VR environment. Furthermore, VR and AR remain new technologies that are not widespread. Hence, we developed a toolbox to capture information within the VR environment and export it in an easy to share format. We developed a dedicated toolbox that features an image capture module within the VR environment and a video capture mode where the user defines landmarks for the displacement of a virtual camera within the virtual point cloud. Furthermore, the user can adjust the timing at each waypoint and set visual parameters such as the color and thresholding. Then, the user can access in desktop mode the newly created external file. The file can be further adjusted manually to define the position of the waypoints and the animation using a dedicated scripting language. In addition, the desktop mode has an interface for timing adjustment. As a showcase, we present the video capture of dual color super-resolution localization microscopy data sets performed in budding yeast (Supplementary Video S3).

#### Advanced Analysis

Point clouds are ubiquitous and thus analysis can vary significantly in complexity (Charles et al., 2017; Qi et al., 2017; Liu et al., 2019). These last 5 years, analysis of point clouds have been the center of numerous efforts in machine learning. The first challenge of any set of point clouds analysis is the data’s variable size, preventing usual machine learning approaches that require data standardization. The second is the complexity of possible underlying models (if they exist) and associated challenges in performing Bayesian analysis (Jaqaman et al., 2008; Höfling and Franosch, 2013). Our domain of application in this paper matches these two challenges. We cannot control time series size, and most underlying models have either no known likelihood formula or are computationally intractable. We refer the reader to (Höfling and Franosch, 2013; Manzo and Garcia-Parajo, 2015; Miller et al., 2017) for a general introduction. Hence, we used simulation-based inference to analyze selected data within the virtual environment. We leverage our recently developed graph neural networks (Fey and Lenssen, 2019; Verdier et al., 2021) to run efficient parameter inferences. We used the following architecture (Supplementary Figure S1, with explication in the caption). The inference model was trained on numerical datasets of various random walk using the “ANDI” package (AnDi, 2020; Muñoz-Gil et al., 2020). The model is pre-trained, and the number of associated parameters is reasonable for a deep model (300,000–500,000). VR representation was done on a GPU card while the inference was run directly on the CPU, leveraging multi-threading to analyze sets of individual trajectories as they were selected by the user.

**FIGURE 4:**
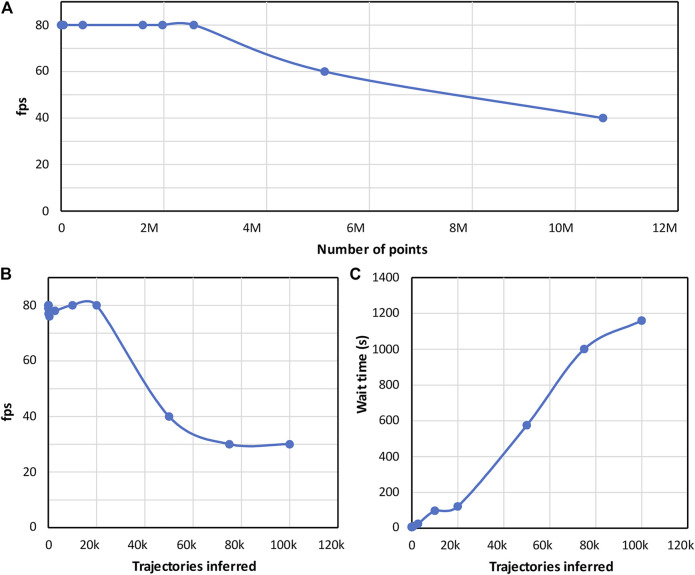
Performance quantification: **(A)** The frame rate of the graphic card is reported as a function of the number of points that are visualized in VR. **(B)** Evolution of the frame rate of the graphic card as a function of the number of the trajectories that are analyzed using the pre-trained neural network. A frame rate of 80 Hz as imposed by the used VR headset defines the range for a comfortable utilization of the VR headset. The refresh rate remains at a comfortable level for up to 30000 trajectories. Such number is considered extremely high compared to realistic data sets. **(C)** Evolution of computation time of the pretrained neural network as a function of the number of inferred trajectories. The reported analysis time is extremely fast regarding the computation required by the GNN. The performance quantifications are performed for a computer with the following specs: Intel Core I5-8400 CPU, 16 Gb ram, Geforce 1080Ti GPU.

In the context of single-molecule tracking experiments, a common task is to find the nature, and associated parameters of dynamical random walks. The underlying idea is that the random walk is a proxy to the physical interactions between the biomolecules and their cellular environments. For this application, typical analysis time is counted in multiple hours. From the localization process of single molecules in images to analysis of trajectories, it can take from hours to multiple days (especially on a single computer). Here, the computation time of the trajectory analysis is massively reduced using a pretrained graph neural network. In our implementation, the challenge was to run an analysis with the pretrained GNN while the representation in VR was running and to analyze the selection of point clouds that could belong to a single or a set of trajectories. Furthermore, the analysis should not significantly slow down the refresh rate to avoid discomfort when analyzing and exploring the data. We found that the rendering performance in Genuage was not impacted when performing the analysis with the pretrained GNN applied on a selection of trajectories. The refresh rate remained at a comfortable rate (Zhang, 2020) of 80 Hz as recommended by the testing VR headset. To test the limits, we quantified the refresh rate as a function of an increasing number of selected trajectories. We found that the refresh rate remained at a comfortable level for selections containing up to 30000 trajectories. Such number is considered extremely high compared to realistic data sets. While the GNN analysis could take up to few minutes to be performed on such large number of trajectories, the VR experience is not impacted, and the user is able to interact with the data during this waiting time. The quantification of the performance is reported in Figure 4.

**FIGURE 5:**
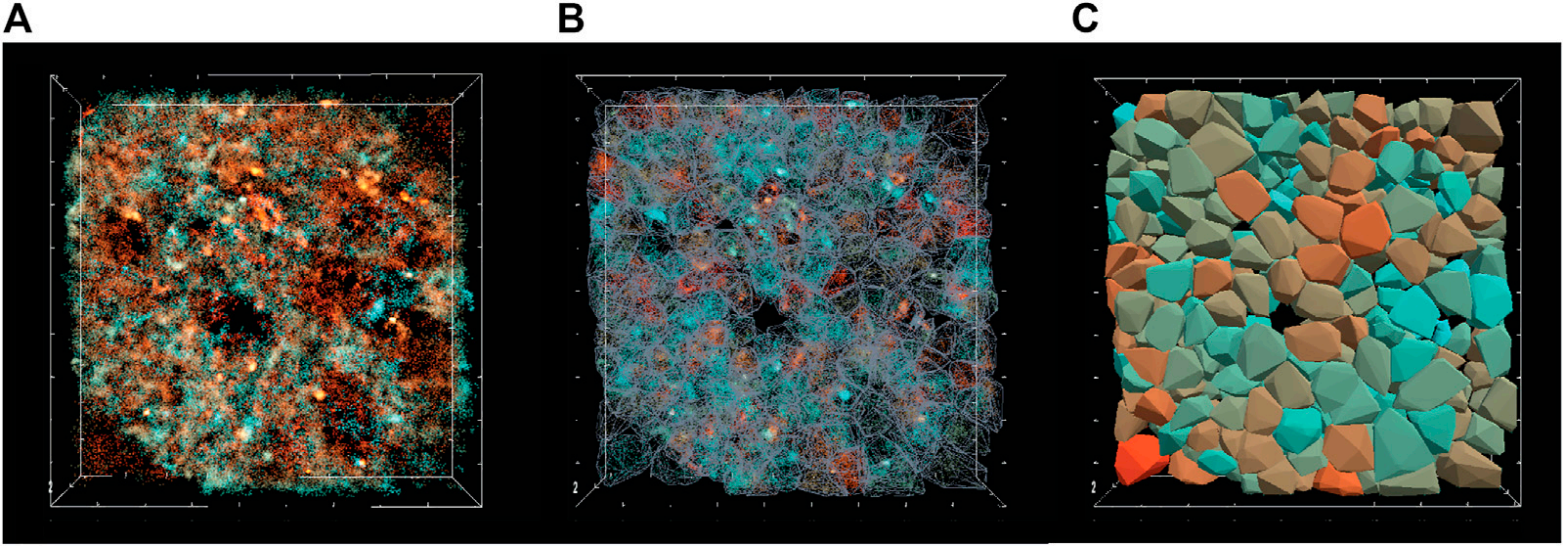
The principle of 3D diffusion map analyses. **(A)** Experimental point cloud data generated from beads injected, imaged in 3D and tracked in the nucleus of a living cell. The panel shows all the recorded localizations color coded by the axial position. **(B)** A 3D mesh allows identifying different regions with a sufficient number of point clouds to perform the analysis. **(C)** in each region, the diffusion coefficient is inferred and shown as a closed surface color coded by the diffusion coefficient.

Nevertheless, when possible, Bayesian Inference remains a powerful analysis tool, especially for mapping spatially physical properties of the cell. One possible analysis approach is determining a landscape of diffusivity and interaction potential based on the recorded point cloud trajectories, including the very short ones, within a given area (Masson et al., 2014; Beheiry et al., 2015) (check supplementary for details). Here we extended this principle to 3D for point cloud data generated thanks to the volumetric imaging capability of MFM. We implemented a 3D mapping of effective diffusion and forces in 3D through a DLL as a proof of concept. First, clusters of point clouds are identified using a k -means clustering algorithm (MacQueen, 1967), and a 3D mesh is generated to define the borders of the different regions. The assumption is that physical properties, such as diffusion coefficient and potential, remain constant in small regions. Second, a Bayesian inference approach estimates the local diffusion coefficient and other physical properties (check supplementary). Finally, the calculated values are uploaded as additional columns. From a visualization point of view, the new challenge here resides in overlaying the generated regions and the associated properties to the point cloud in VR. We opted for a wireframe visualization approach for discriminating the generated 3D mesh and a 3D surface presentation of the associated physical property (Supplementary Video S4). The color code and transparency of each region are adjusted to reflect the calculated physical properties. Figure 5 illustrates the analysis and visualization pipeline.

## Results: Proofs of Concept and Applications

**FIGURE 6:**
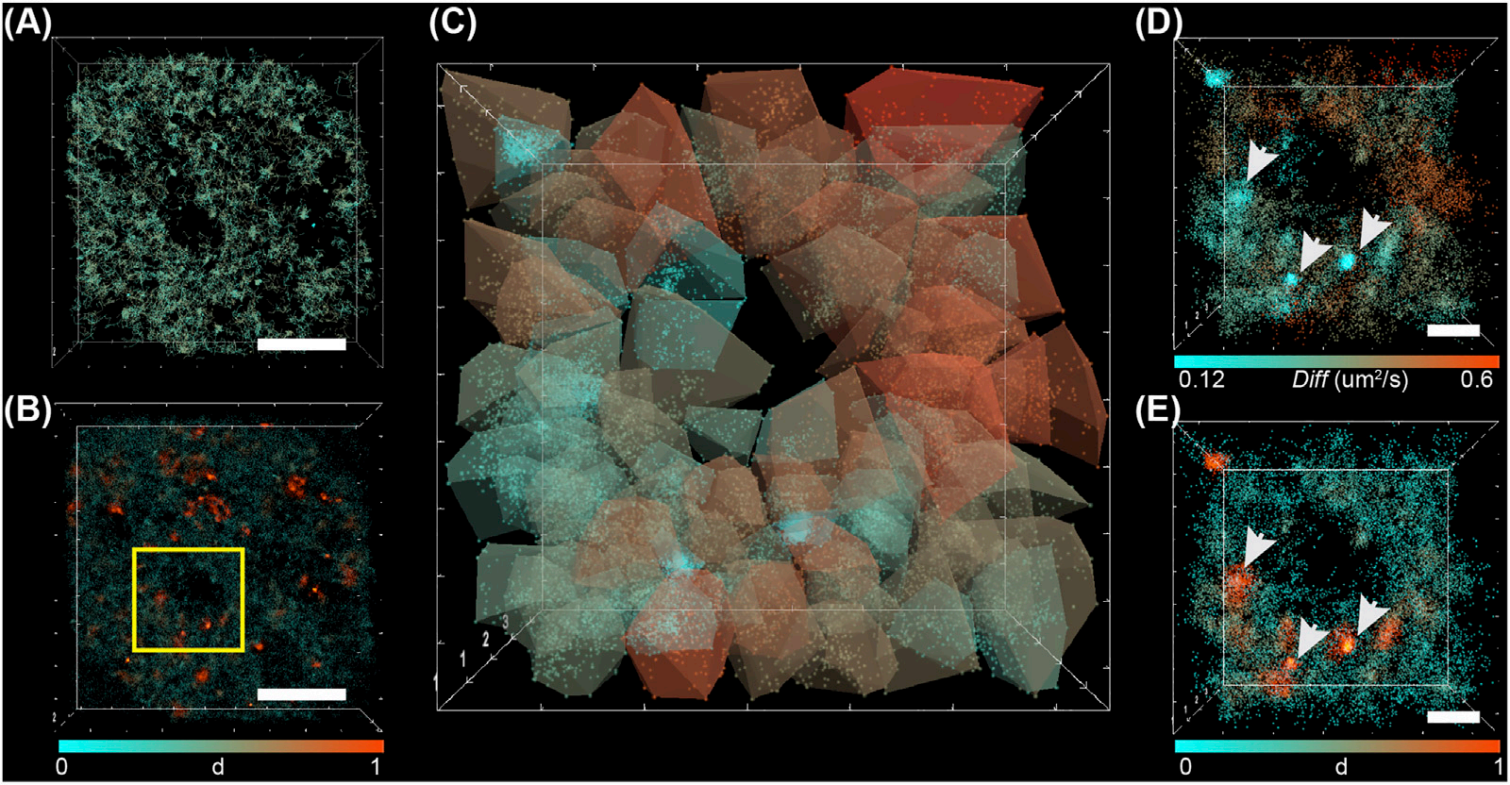
Pipeline for calculating 3D diffusion map of point cloud dynamic data. **(A)** Reconstructed 3D trajectories of beads injected in the nucleus. **(B)** A slice in the reconstructed volume shows a point cloud color-coded by the local density ( d ). A very heterogeneous density is observed pointing towards different chromatin compaction. **(C)** 3D diffusion map generated over a small nucleus area represented by the blue square in **(B)**. The different areas are presented by surfaces with controllable transparency and color-coded by diffusion (*Diff*). **(D)** To easily visualize the diffusion landscape point clouds are colored by the diffusion coefficient. The arrows point toward examples of low diffusivity area where particles are assumed to be trapped. This hypothesis is supported by the corresponding high local density estimation as presented by **(E)**. Scale bar: 5 microns in **(A)** and **(B)**, 1 micron in **(D)**.

Here, we demonstrated our procedures on a set of point cloud data consisting of single-molecule localizations and trajectories of beads injected in the nucleus of living cells. U2OS cells were plated on microscope coverslips. Particles 50 nm in size were injected into the nucleus and allowed to freely diffuse. Volumetric images were recorded at 30 ms exposure time using multifocus microscopy (MFM) (Hajj et al., 2014). Thanks to the addition of several diffractive optical elements on the emission path of a widefield microscope, MFM captures instantaneous volumetric images of the whole cell nucleus by tiling different focal planes side by side on the same camera. MFM fluorescence images are reconstructed as Z-stacks in a post-processing step. The analysis pipeline retrieves the 3D sub-pixel localization of each particle *via* 3D Gaussian fitting. Trajectories are later generated using the Utrack algorithm (Jaqaman et al., 2008) (Figure 2B and Figure 6A). Upon injection, the particles explore the heterogeneous volume of the nucleus, and the diffusive behavior is thought to be a direct indicator of the physical properties and compaction of the surrounding chromatin. In addition, and regardless of the dynamical aspects, the accumulation of all the localizations of the single particles reconstitutes a super-resolution image of the empty space that is surrounded by the chromatin fibers.

In Figure 2 we show a capture of the analysis with the Genuage interface. Supplementary Video S1 shows a full demonstration of the inference being performed sequentially on subsets of selected trajectories. They also show the entire procedure from loading the data to performing a complete analysis of experimental data and procedures to import custom analysis software within Genuage. Analysis time is dependent on the size of the selected data. As a graph is associated with all the selected trajectories, the distribution of trajectory lengths translates into time distributions in receiving the analysis results.

In the Supplementary Figure S6, we show the measured evolution of the framerate with increasing analysis constraints. Beyond 30,000 points, we see a significant decrease in the VR framerate and an increase in the waiting time to receive the analysis result.

Thanks to Genuage and the new set of communication tools, we generated a density map of the particles within the nucleus using an external DLL (provided). It is a direct link to local constraints imposed by the surrounding DNA. Figure 6B shows a slice through the recorded volume where points are color coded by local density. Observed empty spaces are a direct hint for the presence of a chromatin area with high compaction or nuclear compartment that large particles cannot bridge. Conversely, high-density regions point towards trapped particles due to local chromatin density.

To check how compaction affects dynamics and the compaction hypotheses directly linked to diffusion, we analyzed the dynamics of single particles. Real-time analysis allowed us to efficiently explore the complex nuclear environment to retrieve local diffusive properties. As a result, we have observed different diffusion and sub-diffusion exponent parameters in various areas of the nucleus, providing evidence of different compaction levels.

To expand further our investigation, we generated diffusivity maps all over the observed volume and correlated it to the presence of a restricted access area or high particle density area. Figures 6C,D shows a 3D diffusion map generated for a defined area of the nucleus. A very heterogeneous diffusion landscape was observed with intermingled regions of slowly diffusing particles and trapped ones. It is linked to local chromatin compaction and was confirmed by calculating the local density of point cloud Figure 6E.

Future works will focus on disturbing the chromatin organization locally or on a large scale and monitoring the evolution of the diffusion. We think that the VR experience and advanced real-time analysis within Genuage will provide a valuable tool for guiding our future findings.

## Discussion

Virtual and augmented reality will likely play a more critical role in research and medical application. Large scale initiatives using these technologies will fuel the acceptance of the technologies and improve their usability. While initiatives relying on VR to improve complex data visualization are more familiar with applications on microscopy-based data or medical images, we feel that leveraging VR as a part of the analysis pipeline will be a crucial element pushing for research adoption of the tech.

This paper shows dedicated software for real-time visualization and analysis of multidimensional point cloud data such as those generated by single-molecule microscopy. We show that VR is an efficient tool to visualize and interact with complex point-cloud data. Visual features can represent high dimensional data sets and the corresponding output following a quantification process. Genuage is an optimized tool to facilitate data analysis of complex non-isotropic point clouds such as those obtained from biomolecule dynamics.

We have shown a set of libraries and toolboxes implemented in the Genuage platform to perform human-in-the-loop point cloud analysis in VR. Our developments focused on allowing analysis without impacting the image rendering refresh rate and thus ensuring fluid interactions between the user and the data. We provided the required tools to allow efficient data extraction from the VR environment to be displayed onto regular 2D viewing devices. We ensured compatibility with popular analysis frameworks while keeping on adding new ones. Finally, we developed proof of concept analysis pipelines to demonstrate the analysis process in VR and provide guidelines to develop new ones. We chose an application deeply linked to biomolecule research. Nevertheless, the generality of the approach allows applications to any kind of point cloud data.

Our future initiative will center on four topics. First, we will develop Genuage-cloud, a platform extension to allow complex analysis to be run on the cloud through a Django interface and entirely run the visualization and analysis in the cloud while streaming the images to the VR headset. Second, we will extend the Genuage platform to run Augmented Reality (AR) applications on glasses and tablets with a new library focused on amortizing the representation of large point clouds on devices with limited memory. In addition, we will develop new shaders to render the fusion of different data types in the VR environment, facilitating data interpretation during the analysis. Finally, we will develop a physical engine library to allow mixing user interaction and physical simulations to explore organelle structure dynamics in the cell.

## Data Availability

The datasets presented in this study can be found in online repositories. The names of the repository/repositories and accession number(s) can be found below: https://github.com/Genuage/Genuage.
