# Supplementary material for: Towards Human in the Loop Analysis of Complex Point Clouds: Advanced Visualizations, Quantifications, and Communication Features in Virtual Reality
Source: Front Bioinform. 2022 Jan 20;1:775379. doi: 10.3389/fbinf.2021.775379 (PMC9580855; doi:10.3389/fbinf.2021.775379)
Supplement: Supplementary file 1 [file DataSheet1.PDF]

Supplementary Material

1 TCP/IP Communication between Genuage and Python or Matlab processes

Systems using the ZeroMQ framework have been implemented in order to establish communication between Genuage and other processes using the TCP/IP protocol. Thus, it is possible for Genuage to send and receive byte data, Python and Matlab Interfaces are provided. This functionality can be used in two implementations, the first allows users to send numeric data from a MATLAB or python process to Genuage to generate a new cloud, the second is used in the real time inference module, where back and forth communications between Genuage and a python process running a neural network are used to send data for the neural network to infer upon, and get the results back for Genuage to display and save.

2 User-defined DLLs

We provide several DLL examples that are compatible with Genuage to illustrate the different possible input and output formats when performing point cloud quantifications.

The codes are based on Matlab scripts that were transformed into DLLs. The original Matlab files are also provided in the GitHub repository.

The provided DLL are for analyzing single-molecule point-cloud data sets.

- *Density*: The purpose of this DLL is to estimate for each point the number of the neighbors within a given 3D radius. The user inputs the desired radius. The DLL upgrades the raw data set with a new column of the calculated density values.
- *CorrectBlinking*: Analyzing single-molecule localization data sets requires specific considerations especially for super-resolution microscopy data sets. A common artifact arises from the blinking of single molecules. The same molecule can last on multiple frames and thus appears as multiple detected points. The reconstructed image can thus conceal the real underlying molecular distribution. Points arising from the same molecule need to accounted for and combined into single detection.
  This DLL is dedicated for the correction of multiple appearances due to blinking of the same molecule in PALM and STORM imaging. The DLL combines the localizations that are found at a given spatial and temporal proximity. The exact temporal and spatial extents can be estimated by several established methods (Betzig et al., 2006; Rust et al., 2006; Annibale et al., 2011; Coltharp et al., 2012; Puchner et al., 2013; Bohrer et al., 2021). The values are entered manually as numerical input when executing the DLL.
  The code loops over all the frames of the input point-cloud dataset. For each frame, a second loop is performed over a number of frames within search time window (e.g. over 20 frames if the search time is defined at 1 s and the acquisition frame rate is 50 ms). The distances between all the molecules of the considered frame and all the molecules of the following frames are computed. Localizations that can be considered as coming from a same blinking molecule are identified and labelled with an identical index. At the end, the algorithm returns for each localization of each frame its index corresponding to a given unique molecule. Based on this information, a mean position and a mean frame number is computed for each multiple-

appearing molecule. This DLL generates finally a new point cloud with a different number of elements compared to the original one.

- *ApparentDiffusion*: The DLL is dedicated for dynamic point cloud recordings that are generated from single particle tracking experiments. The diffusion of the particles is highly dependent on its physical characteristics (size and functionalization) as well as the surrounding media. As a first order approximation, apparent diffusion coefficient of each trajectory is retrieved from the mean-square displacement (MSD) at different time steps (Normanno et al., 2015; Nora et al., 2020). The apparent diffusion coefficient is estimated from the MSD values that are computed between 2dt$2dt$ and 5dtdt, dt being the exposure time of each acquisition frame. The DLL estimates the diffusion coefficient D of a selected trajectory by a linear fitting of the MSD. The slope is related to the apparent diffusion coefficient by: $slope = 2 \times N \times D$. Here N stands for the dimensionality of the data, e.g. 2 for $2D$ and 3 for $3D$ recordings. The DLL returns the estimated diffusion coefficient of the selected trajectory based on the dimensionality of the data.

We note here that manual inputs are still necessary in some analysis routines, for instance to define the research radius in *Density* and dimensionality in *ApparentDiffusion*. We designed a general interface for providing the necessary numeric arguments (of various types: integers, floats and doubles) for the DLLs.

3 Calculating and presenting physical property map

The 3D maps are generated in a 3-step process.

First, a K-means Clustering algorithm is used to partition the points into a number of clusters based on proximity. The K-means algorithm iterates over a predefined set of starting clusters. Users can set the number of clusters or partitions, as well as the maximum number of iterations for the algorithm to perform. A new data column is added to the cloud at this step, each entry being the id number of the cluster the point belongs to.

Second, Bayesian inference is performed on each cluster of points, using the inference algorithm already included in Genuage (Blanc et al., 2020). Theoretical bases of the inference are described in (Masson et al., 2014; Beheiry et al., 2015). A diffusion coefficient is thus calculated for all the dynamic points within each cluster. User can set various parameters for the priors and the level of noise to be considered by the algorithm. Similarly, a diffusion force vector can be estimated. A new data column is later added to the cloud containing the corresponding diffusion coefficient.

Finally, an implementation of the Quickhull (Barber et al., 1996) algorithm is used to create a 3-dimensional convex hull mesh for the points in each cluster. The resulting meshes can be displayed as solids, colored according to the values of the diffusion coefficient mapped to one of Genuage’s colormaps, or as wireframes. The visibility and opacity of rendered meshes and surfaces can be controlled by the user. The force vectors for the diffusion within each cluster, is displayed as an arrow with a size proportional to the force vector’s norm.

4 3D Map Rendering peculiarities

The meshes for the polygons generated by the 3D physical map module are generated with split vertices, so that no two triangles share the same vertex. This allows for the edges of each face of the polygons to be lit separately by Unity’s lighting engine and for the edges of the solids to be clearly

visible when rendered. The meshes are rendered with a modified version of Unity's standard surface shader, in order to take advantage of Unity's lighting system and be compatible with Genuage's shader-based interactions like the VR Clipping Plane tool.

5 Pretrained neural network

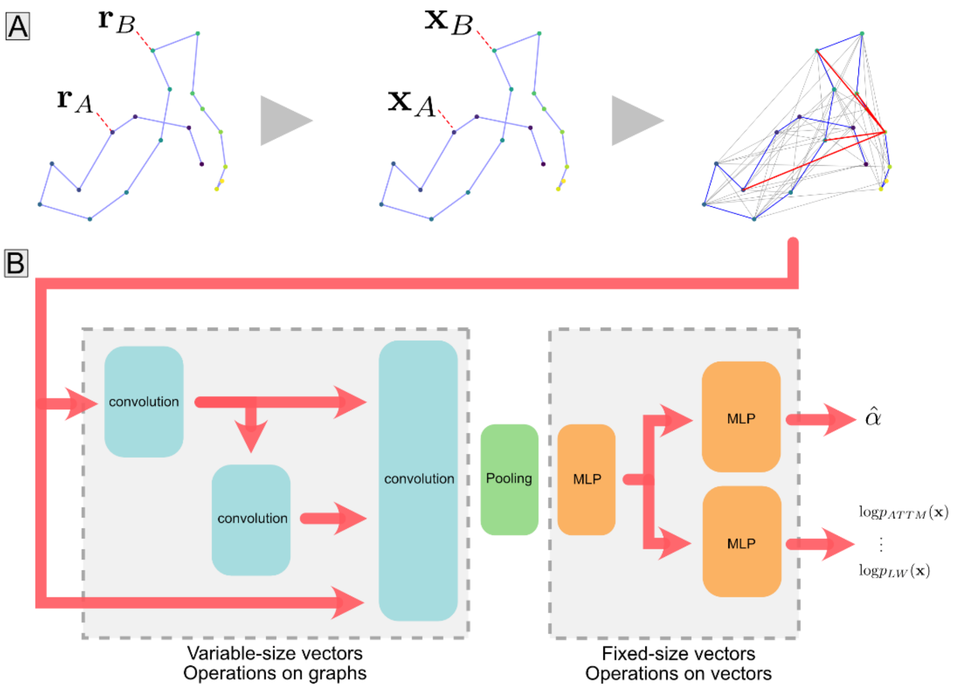

Supplementary Figure 1: a) Construction of a graph associated to a trajectory: a graph is built from successive spatial positions by considering locations (the point cloud) as nodes and by drawing edges between nodes (at random or following a deterministic pattern). Feature vectors are then computed for each node (position, distance to origin, time coordinate, etc.) and for each edge (relative displacement, mean step size of the trajectory between both positions, time difference, etc.). b) Schematic view of a convolution operation: given a "receiving" node (here in red), messages are computed for each neighboring node, from the vector resulting of the concatenation of the neighbor node's features and of the edge features. This vector is given as input to a trainable multi-layer-perceptron (MLP), whose output is a message. After the convolution, each node's feature vector is updated using an aggregation (feature average or maximum) of all the messages it received. The Model architecture: three depths of convolutions are successively concatenated. After the last convolution, features of all nodes are averaged, with an attention mechanism (Knyazev et al., 2019). This averaged feature vector is a latent representation of the trajectory, from which the property of interest (here, the anomalous exponent) is inferred using a classic MLP.

6 Supplementary Figures

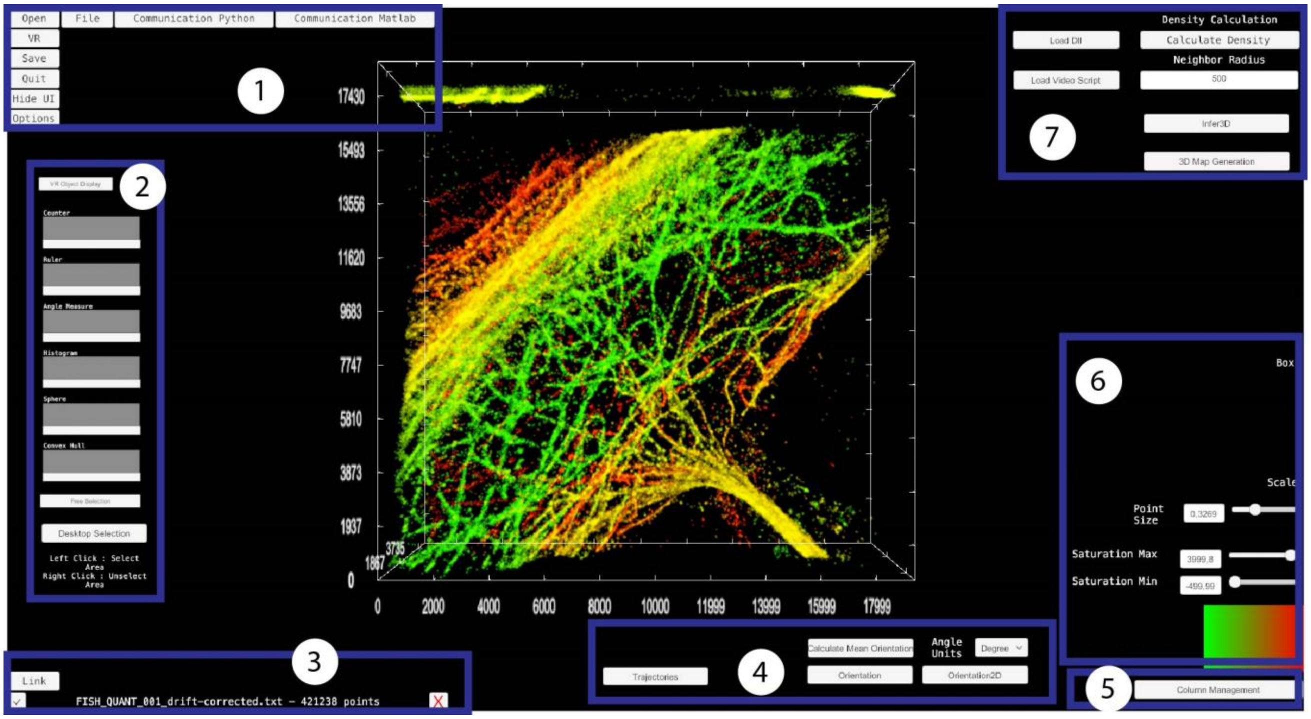

Supplementary Figure 2: General overview of the interface of Genuage in desktop mode. 1: File import and export, communication with external software. 2: objects and selections interface. 3: opened files and multichannel handling. 4: advanced multidimensional data visualization specifically conceived for dynamic data and molecular orientation representation. 5: column management and thresholding. 6: visual parameters control. 7: advanced point cloud analysis.

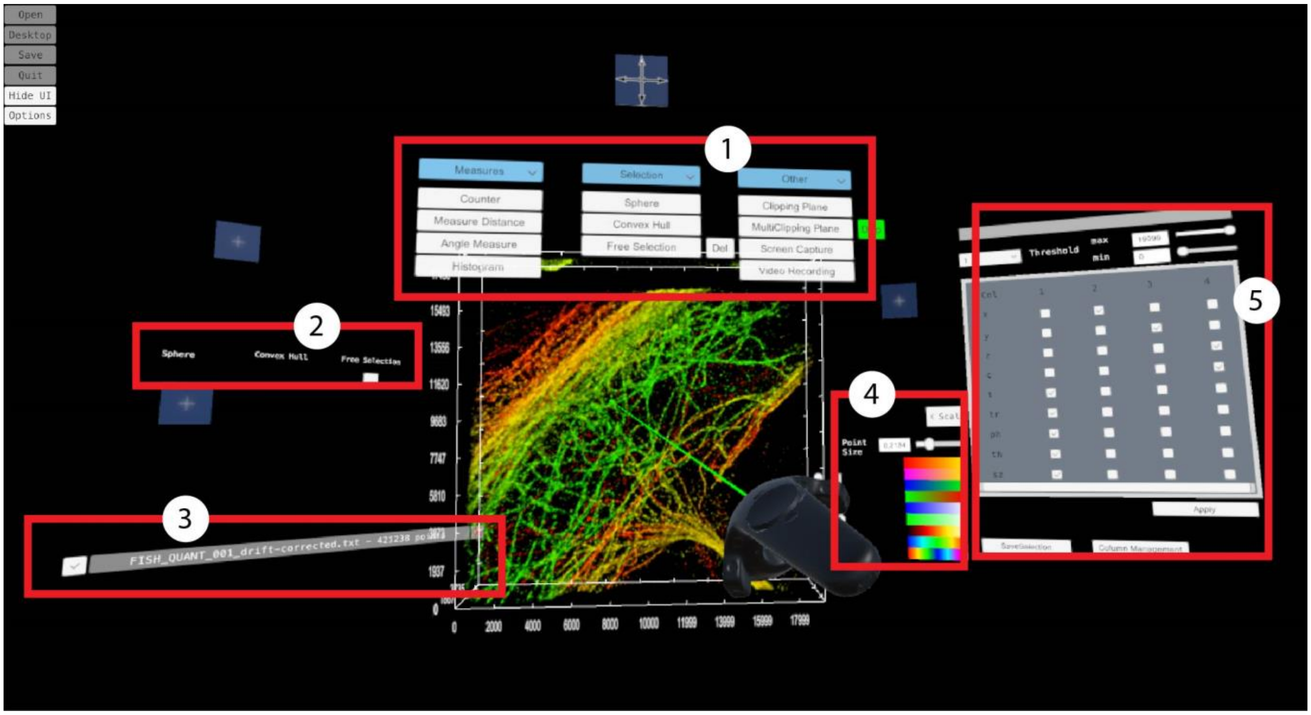

Supplementary Figure 3: General overview of the interface of Genuage in VR mode. 1: A set of tools dedicated to interact and analyze point cloud in the VR mode. It includes (i) performing VR facilitated measurements such as counting, distance, angle and histogram measurements, (ii) specific 3D selections and multi-selections, and (iii) visualization tools such as clipping plane and video recording. 2: Selections and objects interface. 3: Channel interface. 4: Visualization parameters adjustments. 5: Column configuration and thresholding.

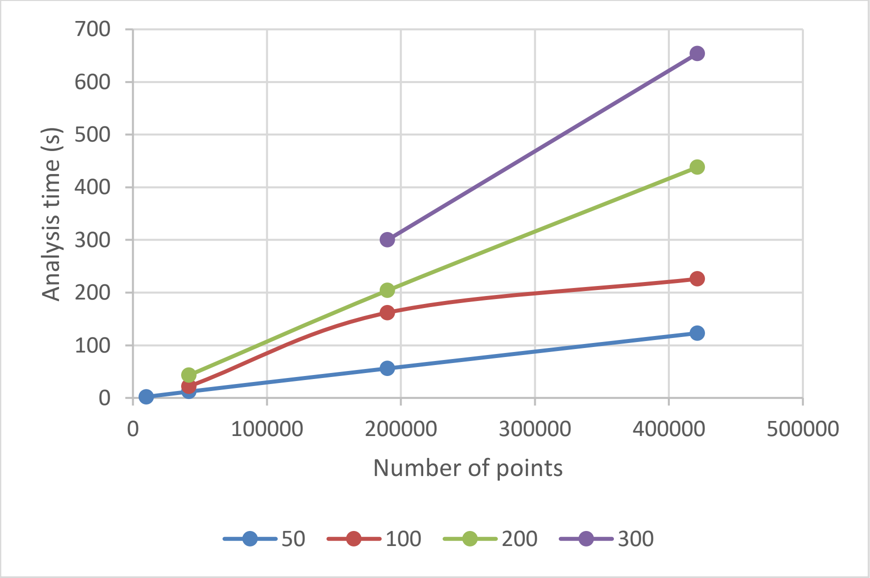

Supplementary Figure 4: Performance characterization of the 3D inference map algorithm as a function of the number of points, evaluated for several number of clusters (50, 100, 200 and 300).

7 Supplementary table

Table comparing different software platforms dedicated to visualizing and analyzing point cloud data. The only relevant comparaison for single-molecule data is between VISP, vLUME and Genuage.

Functionalities		VISP	PointCloud XR	vLUME	CellexalVR	Genuage
**Interface**	Desktop mode	✓	×	×	×	✓
	VR mode	×	✓	✓	✓ yes	✓
	Opening multiple files	✓ into individual tabs	×	✓ in the same working space	×	✓ into individual tabs
	Accepted format	Text files	LAS 1.2 format	csv	✓ Seurat or Scanpy data converted by a custom R package.	Text files
**VR compatibility**	Headsets		HTC Vive, HTC Vive Pro	HTC Vive, HTC Vive Pro Oculus Rift and Rift S / Quest	HTC Vive, Valve Index	HTC Vive, HTC Vive Pro, Oculus Rift and Rift S / Quest
**Visual adjustment**	Color adjustment	✓	✓	✓	✓	✓
	Axis scaling	×	×	✓	✓	✓
	Point cloud size	✓	✓	✓	✓	✓
	Point cloud size by localization precision	✓	×	×		✓
	Multi-color channels	✓	×	✓	✓	✓

**Data input and visualization parameters**	Modular column assignment	×	×	×	×	✓
	Color code assigned to specific column	×	✓	✓	✓	✓
	3D positions	✓	✓	✓	✓	✓
	Trajectories	×	×	✓	✓	✓
	Showing in time	×	✓	✓	✓	✓
	2D Orientation	×	×	×	×	✓
	3D Orientation	×	×	×	×	✓
	Thresholding	✓	×	✓	✓	✓
**VR tools**	Clipping plane	-	×	✓	×	✓
	Freezable Clipping plane	-	×	×	×	✓
	Selections	-	✓	✓	✓	✓
	Counting	-	✓	×	✓	✓
	Measuring distances	✓in desktop mode	✓	×	×	✓
	Histogram profiler	✓in desktop mode	×	×	×	✓
	Annotation in VR	-	×	✓	✓	×
	Data manipulation	-	×	✓	✓	×

	in VR (displacement)					
**Built in analysis tools**	Point cloud local density calculation	✓	×	✓	×	✓
	Ripley's K function	×	×	✓	×	×
	Localization density (in a selection)	×	×	✓	×	×
	Largest and shortest distance	×	×	✓	×	×
	Diffusion coefficient	×	×	×	×	✓ $
	Drift	×	×	×	×	✓ $
	Custom analyses by user made algorithms	×	×	✓through C# scripts	×	✓through C++/Matlab/Other based DLLs
	3D physical properties map generation	×	×	×	×	✓
**Reading/writing**	Exporting videos and figures	✓embeded	✓ Through external software	✓embeded	✓Exporting Images and html reports	✓ embeded
	Saving progress	×	✓	✓	✓ Various saving features	✓ Saving JSON file
	Exporting data	✓	×	✓	✓	✓ Exporting JSON file, exporting selections

	Communication with external software	×	×	×	✓ Communication with R servers	✓

$ Genuage provides up to 9 different methods to calculate the diffusion coefficient, and 10 additional methods to calculate diffusion and drift.

8 Supplementary Videos

Supplementary Video 1: Live dynamic inference performed in Genuage using a pretrained network in Python. The VR experience is not affected by the calculation.
https://youtu.be/ZkG_D0sQlLA

Supplementary Video 2: Examples illustrating the different analysis and possible cloud manipulations in Genuage when running user-defined DLLs.
https://youtu.be/J-WrA3YwJ-8

Supplementary Video 2 bis : A show case for DLL application on a selected trajectory in VR
https://youtu.be/U3bTAy1IVko

Supplementary Video 3: Video recording in Genuage.
https://youtu.be/MWk1Nsg0glA

Supplementary Video 4: Principle of generating 3D diffusion maps in Genuage.
https://youtu.be/VrXVMyuAuCo

9 Supplementary references

Annibale, P., Vanni, S., Scarselli, M., Rothlisberger, U., and Radenovic, A. (2011). Quantitative Photo Activated Localization Microscopy: Unraveling the Effects of Photoblinking. *PLOS ONE* 6, e22678. doi:10.1371/journal.pone.0022678.

Barber, C. B., Dobkin, D. P., and Huhdanpaa, H. (1996). The quickhull algorithm for convex hulls. *ACM Trans. Math. Softw.* 22, 469–483. doi:10.1145/235815.235821.

Beheiry, M. E., Dahan, M., and Masson, J.-B. (2015). InferenceMAP: mapping of single-molecule dynamics with Bayesian inference. *Nat Methods* 12, 594–595. doi:10.1038/nmeth.3441.

Betzig, E., Patterson, G. H., Sougrat, R., Lindwasser, O. W., Olenych, S., Bonifacino, J. S., et al. (2006). Imaging intracellular fluorescent proteins at nanometer resolution. *Science* 313, 1642–1645.

Blanc, T., El Beheiry, M., Caporal, C., Masson, J.-B., and Hajj, B. (2020). Genuage: visualize and analyze multidimensional single-molecule point cloud data in virtual reality. *Nat Methods* 17, 1100–1102. doi:10.1038/s41592-020-0946-1.

Bohrer, C. H., Yang, X., Thakur, S., Weng, X., Tenner, B., McQuillen, R., et al. (2021). A pairwise distance distribution correction (DDC) algorithm to eliminate blinking-caused artifacts in SMLM. *Nat Methods* 18, 669–677. doi:10.1038/s41592-021-01154-y.

Coltharp, C., Kessler, R. P., and Xiao, J. (2012). Accurate Construction of Photoactivated Localization Microscopy (PALM) Images for Quantitative Measurements. *PLOS ONE* 7, e51725. doi:10.1371/journal.pone.0051725.

Knyazev, B., Taylor, G. W., and Amer, M. R. (2019). Understanding Attention and Generalization in Graph Neural Networks. *arXiv:1905.02850 [cs, stat]*. Available at: http://arxiv.org/abs/1905.02850 [Accessed September 9, 2021].

Masson, J.-B., Dionne, P., Salvatico, C., Renner, M., Specht, C. G., Triller, A., et al. (2014). Mapping the Energy and Diffusion Landscapes of Membrane Proteins at the Cell Surface Using High-Density Single-Molecule Imaging and Bayesian Inference: Application to the Multiscale Dynamics of Glycine Receptors in the Neuronal Membrane. *Biophysical Journal* 106, 74–83. doi:10.1016/j.bpj.2013.10.027.

Nora, E. P., Caccianini, L., Fudenberg, G., So, K., Kameswaran, V., Nagle, A., et al. (2020). Molecular basis of CTCF binding polarity in genome folding. *Nat Commun* 11, 5612. doi:10.1038/s41467-020-19283-x.

Normanno, D., Boudarène, L., Dugast-Darzacq, C., Chen, J., Richter, C., Proux, F., et al. (2015). Probing the target search of DNA-binding proteins in mammalian cells using TetR as model searcher. *Nat Commun* 6, 7357. doi:10.1038/ncomms8357.

Puchner, E. M., Walter, J. M., Kasper, R., Huang, B., and Lim, W. A. (2013). Counting molecules in single organelles with superresolution microscopy allows tracking of the endosome maturation trajectory. *PNAS* 110, 16015–16020. doi:10.1073/pnas.1309676110.

Rust, M. J., Bates, M., and Zhuang, X. (2006). Sub-diffraction-limit imaging by stochastic optical reconstruction microscopy (STORM). *Nature methods* 3, 793–796.
